# Dengue virus type 1 clade replacement in recurring homotypic outbreaks

**DOI:** 10.1186/1471-2148-13-213

**Published:** 2013-09-28

**Authors:** Boon-Teong Teoh, Sing-Sin Sam, Kim-Kee Tan, Jefree Johari, Meng-Hooi Shu, Mohammed Bashar Danlami, Juraina Abd-Jamil, NorAziyah MatRahim, Nor Muhammad Mahadi, Sazaly AbuBakar

**Affiliations:** 1 Tropical Infectious Diseases Research and Education Centre (TIDREC), Department of Medical Microbiology, Faculty of Medicine, University of Malaya, Kuala Lumpur 50603, Malaysia; 2 Malaysia Genome Institute, Kajang, Selangor 43000, Malaysia

**Keywords:** Infectious disease, Dengue, Immunity, Malaysia, Evolution, Clade replacement

## Abstract

**Background:**

Recurring dengue outbreaks occur in cyclical pattern in most endemic countries. The recurrences of dengue virus (DENV) infection predispose the population to increased risk of contracting the severe forms of dengue. Understanding the DENV evolutionary mechanism underlying the recurring dengue outbreaks has important implications for epidemic prediction and disease control.

**Results:**

We used a set of viral envelope (E) gene to reconstruct the phylogeny of DENV-1 isolated between the periods of 1987–2011 in Malaysia. Phylogenetic analysis of DENV-1 E gene revealed that genotype I virus clade replacements were associated with the cyclical pattern of major DENV-1 outbreaks in Malaysia. A total of 9 non-conservative amino acid substitutions in the DENV-1 E gene consensus were identified; 4 in domain I, 3 in domain II and 2 in domain III. Selection pressure analyses did not reveal any positively selected codon site within the full length E gene sequences (1485 nt, 495 codons). A total of 183 (mean d*N*/d*S* = 0.0413) negatively selected sites were found within the Malaysian isolates; neither positive nor negative selection was noted for the remaining 312 codons. All the viruses were cross-neutralized by the respective patient sera suggesting no strong support for immunological advantage of any of the amino acid substitutions.

**Conclusion:**

DENV-1 clade replacement is associated with recurrences of major DENV-1 outbreaks in Malaysia. Our findings are consistent with those of other studies that the DENV-1 clade replacement is a stochastic event independent of positive selection.

## Background

Dengue virus (DENV) is a virus of the *Flaviviridae* family. It is an enveloped positive-sense single-stranded RNA arbovirus with a genome of approximately 11 kb [1]. There are four antigenically distinct DENV serotypes; DENV-1, DENV-2, DENV-3 and DENV-4 [2] and each serotype shows phylogenetically distinct genotypes [3]. The virus is transmitted to susceptible hosts through bites of infected mosquitoes. The virus is maintained in sylvatic nonhuman primate/sylvatic mosquitoes and endemic human/urban/peridomestic mosquitoes cycles. All four DENV serotypes are believed to have independently evolved from separate sylvatic ancestral lineages through either peridomestic/urban mosquitoes or human hosts 100–1,500 years ago [4]. Currently, an estimated 3.6 billion persons living in dengue-endemic countries are at risk of contracting dengue. The number of countries reporting dengue has in recent years escalated to more than 125 suggesting successful adaptation and dissemination of the virus [5].

In dengue endemic regions, heterotypic and homotypic major dengue outbreaks occur in cyclical patterns of approximately every 3–5 years and 7–10 years, respectively [6-10]. The major concern associated with recurring dengue outbreak in endemic countries is the risk of contracting the severe forms of dengue especially following second infection with a heterotypic virus [11]. Antibody-dependent enhancement [12,13], original antigenic sin [14,15], cytokine storm [16], and autoimmune responses [17,18] are the possible mechanisms contributing to the manifestation of severe dengue. It has been reported that infection with one DENV serotype confers lifelong protection against homotypic reinfection but only temporary cross-protection against heterotypic infection [19]. The presence of sub-neutralizing and cross-reacting antibodies is suggested to play important role in the manifestation of the severe dengue [20,21]. Reports of repeated infection with dengue is however, not uncommon in dengue endemic regions [22,23]. Understanding the factors contributing to the recurrence of dengue outbreaks has important implications for our understanding of dengue epidemiology. Knowledge gained from this understanding could help improve dengue surveillance and outbreak prediction and preparation. It could also help to facilitate selection of better dengue vaccine candidates.

Earlier studies have suggested that DENV clade replacement is linked to the recurring and cyclical pattern of dengue outbreaks in many endemic countries [6,24-27]. From these studies, it is suggested that clade replacement is associated with positive selection due to the differences in viral fitness between clades; new virus with a higher viremia level in human [9] or enhanced infectivity to mosquito [28-31] could be positively selected to replace the old virus which was less fit. In contrary, several other phylogenetic studies of DENV have suggested that the clade replacement is solely a stochastic event due to the virus population bottleneck effects [24,25]. Although the potential mechanisms of DENV evolution underlying the clade replacement have been investigated, correlation studies involving the host immunological factors have not been adequately addressed. Zhang *et al.* proposed that the DENV-1 clade replacement is associated with the cross-protective immunity accorded by DENV-4 based on a longitudinal dengue epidemiological study in Bangkok [6]. Whereas Adams *et al.* employed a mathematical model to demonstrate that the degree of interserotypic cross-protective immunity could account for the cyclical pattern of heterotypic outbreak in Bangkok [32].

In the present study, we used the recurring DENV-1 outbreaks in Malaysia which occurred in 1987, 1997 and 2004 [8,33], as a study model. The availability of serially collected DENV-1 since 1987 within a single locality (Klang Valley) provides us with an opportunity to explore the temporal phylogenetic evolution that shapes the virus clade replacement in recurring DENV-1 outbreaks in Malaysia. We used DENV-1 full length envelope (E) gene sequence to reconstruct the phylogeny and investigated the presence of homotypic cross-neutralizing antibody of patients with primary DENV-1 infection.

## Results and discussion

**Figure 1:**
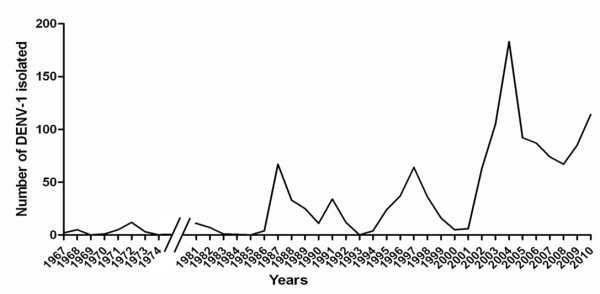
**DENV-1 isolated in Malaysia for the period 1967–2010.** The number of DENV-1 isolated for the period 1975–1980 is not available.

**Figure 2:**
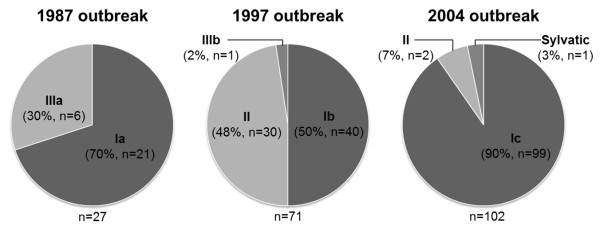
**Distribution of DENV-1 genotypes in recurring DENV-1 outbreaks in Malaysia.** The numbers below the pie charts indicate the total numbers of DENV-1 used from each outbreak. Total numbers of DENV-1 used for 1987, 1997 and 2004 outbreaks included viruses for the period 1987–1989, 1997–1999 and 2004–2006, respectively.

In the study, a total of 335 Malaysian DENV-1 isolates collected from 1987 to 2011 were used. All viruses were isolated from dengue patients living in single locality, Klang Valley. Between the periods, three major DENV-1 outbreaks occurred in 1987, 1997 and 2004 with a cyclical pattern of ~8 years interval (Figure 1) [8,33]. Preliminary phylogenetic analysis performed using the full E gene sequence revealed that the virus collection comprised of DENV-1 from genotype I, II, III and sylvatic group with frequency of 80.6% (n = 270), 16.1% (n = 54), 3.0% (n = 10) and 0.3% (n = 1), respectively (data not shown), suggesting that DENV-1 outbreaks in Malaysia over the last 3 decades were mainly caused by the genotype I and II viruses. Figure 2 summarized the relative percentages of DENV-1 genotypes isolated during the three major outbreaks. Due to the large number of DENV-1 available for each outbreak, the viral sequences with >98% nucleotide identity within each monophyletic group were reduced to 3–6 sequences. A total of 44 representative isolates that covered all possible viral genetic diversity over time were presented in this study.

**Figure 3:**
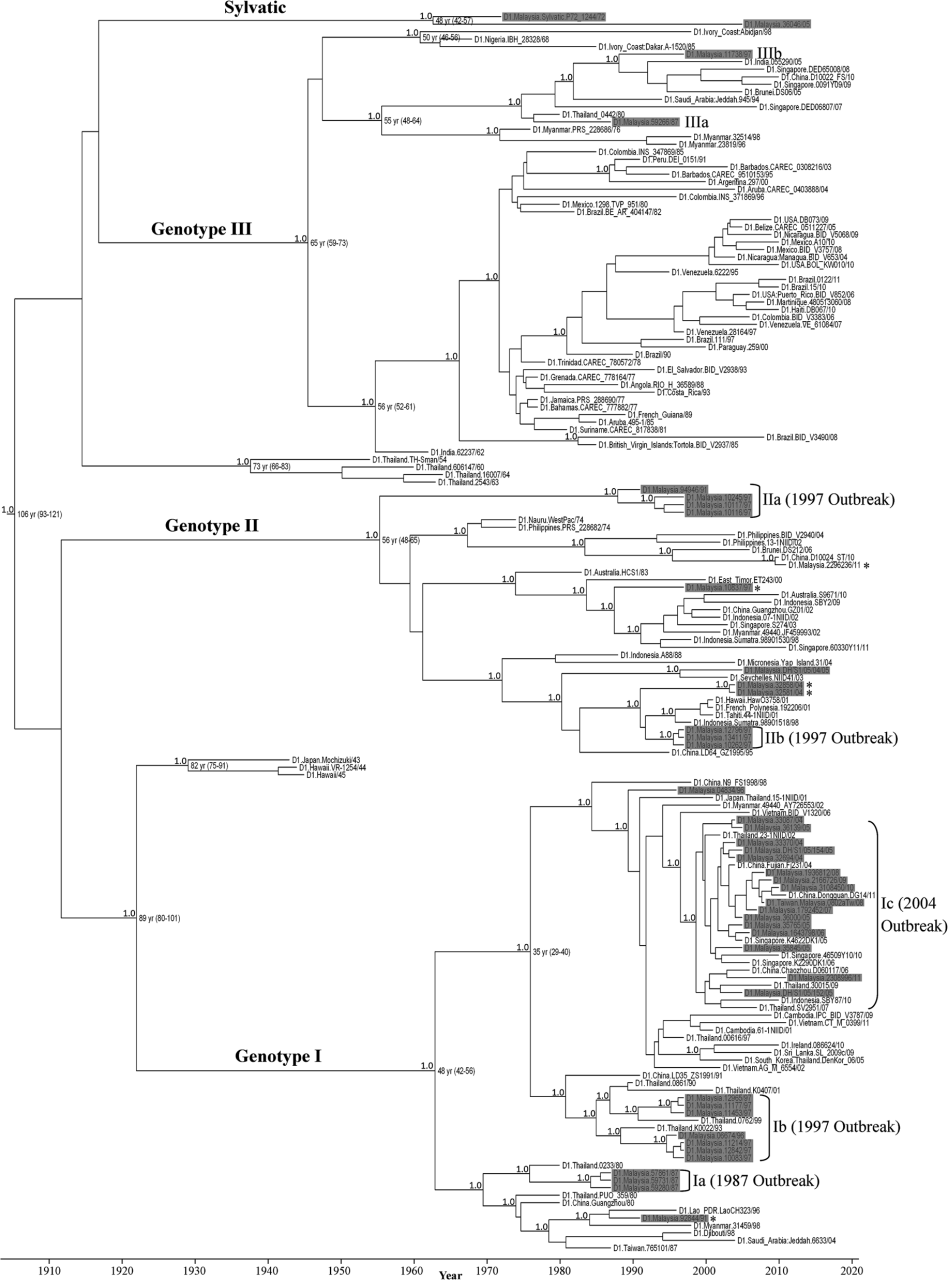
**Maximum clade credibility tree of complete envelope genes of DENV-1.** Horizontal branches are drawn to a scale of estimated year of divergence. Coalescent times with 95% highest posterior density values (ranges in parentheses) and posterior probability values (all 1.0) of key nodes are shown. DENV-1 outbreaks are indicated at the end of branches according to the outbreak-causing clades. The consensus E amino acid sequences of Malaysian DENV-1 isolates used were indicated as Ia, Ib, Ic, IIa, IIb, IIIa and IIIb. The DENV-1 isolates from Malaysia are highlighted in grey. Viruses marked with * are the rare isolates. A total of 39 new Malaysian DENV-1 E gene sequences were used [EMBL:FN825674, EMBL:FR666920-FR666928].

The Bayesian phylogenetic tree constructed using the full E gene sequence (1485 nt) revealed the existence of at least six distinct genotypes (Figure 3): three ancestral genotypes (Japan/Hawaii 1943–1945, Thailand 1954–1964 and Malaysia/sylvatic 1972–2005) and three endemic/epidemic genotypes (genotype I, II and III) in concordance to the previous classification method [33,34]. DENV-1 genotype I viruses were involved in outbreaks in 1987, 1997 and 2004, while genotype II viruses were only involved in the 1997 outbreak.

Genotype I comprised mainly of the Asian strains with the exception of an African strain (D1.Djibouti/98). The genotype I viruses from the 1987, 1997 and 2004 outbreaks grouped into clade Ia, Ib and Ic, respectively in a ladder-like topology (Figure 3) suggesting temporal clade replacement occurred in each of the major DENV-1 outbreak. All the genotype I clades consisted of Thai viruses with approximately similar isolation years (±7 years), suggesting that the same pool of DENV-1 strains circulated within Thailand [6]. Viruses in clade Ia and Ib shared clear ancestral lineages with Thai isolates whereas those forming clade Ic could have evolved from another Malaysian isolate seen in 1996 (D1.Malaysia.04834/96). The clade Ic viruses caused the largest major DENV-1 outbreak in Malaysia in 2004 and the viruses shared high sequence similarity to isolates recovered from a major outbreak in Singapore in 2005 [35]. The clade Ic viruses were recovered much earlier in Thailand (1997), China (1998), Cambodia (2001), Vietnam (2002) and Myanmar (2002) before it caused outbreak in Malaysia and Singapore. Though all the clade Ic viruses shared common ancestral lineage to the Malaysian 1996 isolate, it is possible that the Malaysian 1996 virus was endemic in Malaysia but remained in the background until it achieved fitness to cause outbreak later in 2004 [33]. This fit the expected homotypic cycle of major dengue outbreak in Malaysia at approximately every 8 years. Since the 2004 outbreak, clade Ic viruses were continuously isolated in Malaysia, Singapore, Thailand, Vietnam, Cambodia and China until 2011 and no clade replacement event of DENV-1 was observed then. The magnitude of 2004 outbreak involving many Asian countries could probably extent the time interval between the occurrences of major homotypic DENV-1 outbreaks in the future. To date, the clade Ic viruses have been imported into Japan (2001), South Korea (2005), Taiwan (2008), Sri Lanka (2009), Indonesia (2010) and Ireland (2010) [36-39] suggesting a possible global spread of the virus to other parts of the world. This could represent the most successful distribution of DENV-1 to date akin to that observed for the Cosmopolitan DENV-2 [40]. On the other hand, the persistence of a ladder-like phylogenetic tree topology predicts possible emergence of a clade Id DENV-1 in the next major DENV-1 outbreak in Malaysia perhaps in or around 2019.

The genotype II viruses obtained in the study shared high sequence similarities to viruses from a wider geographical distribution including Asia and Pacific Ocean regions with the exception of an Indian Ocean strain (D1.Seychelles.NIID41/03). In addition to clade Ib, genotype II viruses (clade IIa and IIb) were also recovered during the 1997 outbreak. Phylogenetic analysis suggests that the clade IIa viruses could have evolved from another Malaysian isolate seen in 1991 (D1.Malaysia.94946/91). The clade IIa viruses are likely the indigenous DENV-1 of Malaysia as no virus from other country was found within the clade. In contrast, clade IIb viruses grouped with isolates from Indonesia and South Pacific islands including Micronesia and Polynesia. All the clade IIb viruses shared common ancestral lineage to the Indonesia 1988 isolate. The presence of the Indonesian isolates during the 1997 outbreak could be due to importation of the viruses through influx of migrant workers as there was a surge of workers from Indonesia to Malaysia during that time [41]. In 2004, two isolates, D1.Malaysia.32858/04 and D1.Malaysia.32581/04, which shared high sequence similarities to the clade IIb viruses were recovered. This could be either another incidence of importation of the Indonesian isolate or an *in situ* evolution of the clade IIb viruses. The importations of DENV-1 strains from the neighboring countries would increase the genetic diversity of DENV-1 in Malaysia.

Genotype III consisted of mainly DENV-1 isolates from Latin America. Asian and African strains of the genotype III, however, have been occasionally isolated. The genotype III viruses from Malaysia, D1.Malaysia.59266/87 and D1.Malaysia.11738/97, grouped with isolates from Saudi Arabia, Brunei, India, Singapore and China and shared common ancestral lineage to the Thai 1980 isolate. In our samples, only 10 genotype III isolates (6 in 1987, 2 in 1995, 1 in 1996 and 1 in 1997) were recovered from patients during the period 1987–1997. The virus has not been isolated since 1998 which suggests its possible extinction from Malaysia. Similar findings were observed in Thailand and Myanmar in 1983 and 1998, respectively. The lineage extinction of genotype III was hypothesized to be due to a stochastic event attributable to possible low rate of virus transmission during an inter-epidemic period [6,42]. However, the emergence of genotype III virus in Singapore in 2007 suggests possible reintroduction of genotype III virus to Malaysia in the future [43].

**Figure 4:**
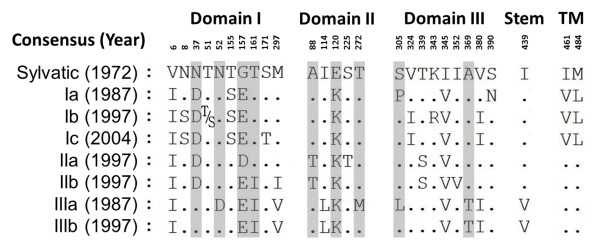
**Observed amino acid substitutions in Malaysian DENV-1 E gene consensuses.** The non-conservative amino acid substitutions which resulted in the polarity changes are highlighted in grey. TM = Transmembrane.

Using the ancestral sylvatic virus E amino acid sequence (D1.Malaysia.Sylvatic.P72_1244/72) as the basal sequence, the consensus amino acid sequences of all the Malaysian isolates were found highly conserved, with identities ranging from 96 to 98% between the genotypes (Figure 4). Although the genotype Japan/Hawaii 1943–1945 viruses served as the common ancestral lineage for all the genotype I viruses (Figure 3), the identities of consensus amino acid sequences between sylvatic virus and genotype I viruses were found to be higher (97%) than those between the Japan/Hawaii 1943–1945 viruses and genotype I viruses (ranging from 95 to 97%) (data not shown). Three conserved yet functionally important regions of DENV-1 E were observed: the twelve disulfide bond-forming cysteine residues; the glycine-rich fusion domain (residues 98–111); and two glycosylated asparagines at position Asn-67 and Asn-153 (data not shown) [44-46]. Out of 27 amino acid substitutions observed, only 9 (highlighted in grey) resulted in polarity changes (non-conservative amino acid substitutions). These non-conservative substitutions were found mostly in domain I (residues 37, 52, 157 and 161), followed by domain II (residues 88, 120 and 272) and domain III (residues 305 and 369), suggesting that domain I may be less functionally critical than domain II and domain III [47]. The individual functional effect of each conservative amino acid substitution may be unnoticeable or relatively smaller than those of non-conservative substitutions, but the cumulative effects of two or more neighboring conservative substitutions at the three-dimensional space could be important for the proper protein function.

In this study, we noted that the non-conservative amino acid substitutions E120K (domain II) and G157E/D (domain I) differentiated the three endemic genotypes from the ancestral sylvatic group. Several other amino acid substitutions gave rise to additional three endemic genotypes (Figure 4). Within genotype I, two non-conservative amino acid substitutions P305S (domain III) and N390S (domain III) were identified in the 1997 viruses when compared against the 1987 viruses. No non-conservative amino acid substitution was observed between the viruses from 1997 and 2004 outbreaks. The decreasing number of amino acid substitution among the genotype I viruses from 1987 to 2004 could suggest that the viruses may have reached a point where all of the non-conservative amino acid substitutions were deleterious.

Within genotype II, two non-conservative amino acid substitutions D157E and T161I in domain I further differentiated the viruses into the clade IIa and IIb. Temporal analysis of amino acid substitution was not performed for the clade IIa and IIb viruses due to their similar emergence time during the 1997 outbreak. Whereas within genotype III, two non-conservative amino acid substitutions L305S (domain III) and M272T (domain II) were found between clade IIIa and IIIb. The M272T substitution located at the “kl” *β*-hairpin region (residues 270 to 279). This “kl” *β*-hairpin is a pH-dependent hinge region involved in the fusion-activating conformational change [44]. Therefore, any non-synonymous mutation in the “kl” *β*-hairpin region could affect virus replication by altering the fusion pH threshold resulting in inefficient virus replication [48,49]. This could help explain the rapid extinction of the genotype III virus as the virus could not be efficiently propagated.

Selection pressure analyses of viral E gene collectively found 183 (mean d*N*/d*S* = 0.0413), 136 (mean d*N*/d*S* = 0.0852), 245 (mean d*N*/d*S* = 0.0559) and 196 (mean d*N*/d*S* = 0.0704) negatively selected sites within the Malaysian isolates, the genotype I, II and III isolates, respectively. No positively selected codon site was noted within the Malaysian isolates and within each of the genotypes. All observed amino acid substitutions in Malaysian DENV-1 E gene consensuses (Figure 4) were found to be under negative selection except at the codon 8, 52, 161, 272, 339 and 390 which had no evidence of either negative or positive selection but probably happened through neutral genetic drift. Based on the Bayesian inference, the evolutionary rates of DENV-1 E gene were estimated to be 3.39 × 10^-4^-14.64 × 10^-4^ substitutions/site/year. The evolutionary rates of DENV-1 E gene did not differ substantially from those of other DENV serotypes (5.42 × 10^-4^-11.58 × 10^-4^ substitutions/site/year) [50]. These rates, however, were relatively lower than those of non-vector-borne RNA viruses (~10^-3^ substitutions/site/year) such as influenza viruses and HIV [51]. The DENV-1 evolution in Malaysia was probably constrained by purifying selection [52].

The cross-neutralizing capacity of convalescent patient serum samples from 1997 (Ib, IIa and IIb) and 2004 (Ic) outbreaks were evaluated against viruses from seven clades: Ia (D1.Malaysia.59280/87), Ib (D1.Malaysia.11177/97), Ic (D1.Malaysia.36000/05), IIa (D1.Malaysia.11708/97), IIb (D1.Malaysia.12796/97), IIIa (D1.Malaysia.59266/87) and sylvatic group (D1.Malaysia.36046/05) (Table 1). The neutralization titers of Ib sera against genotype I viruses (FRNT_80_ = 320–1280) were higher than those of Ic, IIa and IIb sera (FRNT_80_ = 80–320). All sera had FRNT_80_ = 80 against genotype II and IIIa viruses except Ic sera, which showed FRNT_80_ = 320 against the genotype IIb and IIIa viruses. The neutralization titers of the IIa sera against sylvatic virus (FRNT_80_ = 80) were lower than those of Ib, Ic and IIb sera (FRNT_80_ = 320). Overall, all viruses were neutralized by pooled sera from the 1997 and 2004 outbreaks with neutralization titers ranging from FRNT_80_ = 80–1280. Interserotypic interaction of DENV-1 patient’s sera with other DENV serotypes, however, is not available in this study to rule out any possible cross-reactivity among them. Results presented here suggested that there was no apparent immunological advantage accorded by the new amino acid substitutions for the virus in countering the homotypic human herd immunity. Therefore, the availability of a new susceptible human population may be obligatory for DENV-1 to initiate a new outbreak.

**Table 1:** **Neutralization of DENV-1 from the different clades**^**a **^**using the respective DENV-1 patient serum**

	**Virus clades**						
**DENV-1 patient serum**^**b**^	**Ia**	**Ib**	**Ic**	**IIa**	**IIb**	**IIIa**	**Sylvatic**
Mock^c^	0	0	0	0	0	0	0
Ib	320	320	1280	80	80	80	320
Ic	320	80	320	80	320	320	320
IIa	320	80	320	80	80	80	80
IIb	80	320	320	80	80	80	320

^a^ Neutralization titer was defined as an 80% reduction in the foci reduction neutralization test (FRNT_80_).

^b^ Serum sample from clade Ia is not available.

^c^ Control treated with serum from healthy (no dengue infection) donors.

The human herd immunity developed after the DENV-1 outbreak could suppress the horizontal transmission of virus to human population by infected mosquito [19]. This could restrict the virus to continue to exist only in mosquito population through vertical transmission [53,54]. The population dynamics of mosquito over time, however, are mainly fluctuated by the stochastic environmental factors and the intermittent vector control measures [55,56]. This could repeatedly cause virus population bottlenecks (small population size) which favor the amino acid substitutions by genetic drift over those by natural selection [57]. Even if the positively selected substitutions are advantageous, it could be randomly lost over time due to the bottleneck effect. By contrast, the neutral substitutions could be easily fixed in small virus population over time by chance [58]. In our study, six codons with observed amino acid substitutions in DENV-1 E gene consensuses were under neutral genetic drift.

Our results suggest that the DENV-1 evolution in recurring outbreak in Malaysia is likely to be a stochastic phenomenon, possibly driven by both negative selection and genetic drift [6,24,25,52]. Whether the observed amino acid substitutions would cause enhanced virus replication in mosquito or human requires further investigation. During the inter-epidemic period, the increased viral fitness, however, may have no potential epidemic impact due to the human herd immunity that broadly cross-neutralized among different DENV-1 genotypes. Phylogenetic analysis in this study revealed that the emergences of clade IIb (1997 outbreak) and clade Ic viruses (2004 outbreak) were detected as early as in 1991 (D1.Malaysia.94946/91) and 1996 (D1.Malaysia.04834/96), respectively. The reason why these viruses did not cause outbreak when they were first encountered could be due to the presence in the population of cross-neutralizing immunity accorded by other DENV-1 genotypes. The cyclical pattern of recurring DENV-1 outbreaks in Malaysia suggests that a lag period of 7–10 years after the old DENV-1 outbreak would be required for a newly evolved virus to initiate another homotypic outbreak. During this period, an increasing number of new susceptible human hosts may gradually break down the human herd immunity below the protective threshold, thus allowing the restoration of virus horizontal transmission cycle in human population. This suggests the possibility that it is not always the fittest, but the fortuitous DENV-1 would be selected to be the clade replacement candidate which would initiate the next outbreak. And this could occur when the ecological situation favors the virus transmission. The appearances of rare isolates such as D1.Malaysia.92844/91, D1.Malaysia.10837/97, D1.Malaysia.32581/04, D1.Malaysia.32858/04 and D1.Malaysia.296236/11 (Figure 3, marked with *) could represent the potential clade replacement candidates for the future major outbreaks.

The present study is limited by working only with the viral E gene, such that evolutionary pressures on other gene regions will be missed. Also, during the inter-epidemic periods between the homotypic outbreaks, the available sample set selected for sequencing is limited. This leaves gaps in understanding the extinction and emergence of the different clades. Thirdly, the interserotypic interaction between the DENV-1 with other co-circulating virus serotypes is not addressed in this study. This is important as the interserotypic immune reaction could account for the alternating epidemic pattern of DENV serotypes in endemic regions. The cross-neutralization assay in this study is within the limitation where the possible cross-reactivity between different DENV serotypes was not examined. This was due to the unavailability of sufficient convalescent sera from dengue patients since the study was using retrospectively collected sera. As such further studies which will include early convalescent serum from DENV-2, DENV-3 or DENV-4 infection is desirable. Another limitation of the present study is that the immune responses of patients were averaged in the pooled sera; the neutralizing capacity of each serum might vary depending on the time of blood collection and genetic background of patients. Notwithstanding the limitation of the study, findings from the study provide opportunities to understand the possible mechanisms driving to cyclical pattern of major dengue outbreaks in endemic regions.

## Conclusions

DENV-1 clade replacement is associated with the recurring major DENV-1 outbreaks in Malaysia. Our findings from selection pressure analyses and neutralization assays are consistent with earlier studies [24,25] suggesting that virus clade replacement is stochastic rather than driven by positive selection.

## Methods

### Viruses

The DENV-1 isolates used in this study were isolated from Klang Valley during the period of time spanning 25 years (1987–2011). Almost all the isolates came from patients who seeked medical treatment at the University Malaya Medical Centre (UMMC), a major referral hospital serving an estimated of 8.1 million people, mostly living within 25 km of the hospital. All the viruses were archived at the UMMC Diagnostic Virology Laboratory. The isolates at passage 1 were used to inoculate C6/36 (*Aedes albopictus*) mosquito cells for one week and virus RNA was extracted from this infected cell culture supernatant.

### RNA extraction, RT-PCR and nucleotide sequencing

Viral RNA was extracted using QIAamp Viral RNA Mini Kit (Qiagen, Germany) strictly following the manufacturer’s instructions. One-step RT-PCR was performed to amplify DENV-1 E gene using the primers as previously described [34]. The amplified DNA fragments were purified using QIAquick Gel Extraction Kit (Qiagen, Germany) and sequenced using the BigDye Terminator v3.1 Cycle Sequencing Kit on an automated capillary DNA sequencer 3730xl DNA Analyzer (Applied Biosystems, USA).

### Phylogenetic analysis

The DENV-1 E gene dataset for phylogenetic analysis comprised of 44 isolates from Malaysia and 121 global sequences representing the 6 distinct DENV-1 genotypes as previously reported [33,34]. The maximum clade credibility tree (MCC) was inferred by using the Bayesian Markov Chain Monte Carlo (MCMC) method implemented in BEAST version 1.6.2 [59]. The best-fit model of nucleotide substitution was selected by Akaike Information Criterion (AIC) and Bayesian Information Criterion (BIC) as implemented in jModelTest 0.1.1 [60]. Both AIC and BIC approaches found the TN93 + G_4_ + I model as the best-fit model for the dataset. Two independent MCMC analyses (100 million steps) were performed utilizing relaxed uncorrelated lognormal molecular clock with Bayesian skyline as coalescent prior. The convergence of the chain was evaluated by using Tracer 1.5 [59]. The effective sample size (ESS) values of >200 indicated sufficient level of sampling. The MCC tree was generated and visualized by using TreeAnnotator program and FigTree 1.2.3, respectively. The degree of uncertainty in each parameter estimate is provided by the 95% highest posterior density (HPD) values.

### Selection pressure analysis

The selection pressure analysis was performed by using the online facility of Datamonkey web server (http://www.datamonkey.org) based on the neighbor joining tree [61]. Four datasets were included, one containing only Malaysian isolates (n = 44) and the other three containing isolates belonged to the three different virus groups, genotype I (n = 63), genotype II (n = 36) and genotype III (n = 57). Five likelihood-based algorithms: SLAC, FEL, IFEL, REL and PARRIS methods were used to identify the existence of positive selection pressure at individual codon sites in the DENV-1 E gene. Sites were considered to be under positive selection if the ratio of non-synonymous (d*N*) to synonymous (d*S*) substitutions per site (ratio d*N*/d*S*) indicated with high statistical significance (P <0.1 / Bayes factor >50).

### Patient sera

The study obtained ethics approval from the UMMC Medical Ethics Committee (Ethics Committee/IRB Reference Number: 860.24 and 908.9). Paired (acute and convalescent) DENV-1 patient serum samples from 1997 (Ib, IIa and IIb) and 2004 (Ic) outbreaks were obtained from UMMC Diagnostic Virology Laboratory. Informed consents were not obtained from the patients as this study was using retrospectively collected sera. The acute and convalescent serum samples were taken at a mean of 4.3 days (range, 1–6 days) and 12.5 days (range, 5–24 days) post-onset, respectively. Acute DENV-1 infection was confirmed by virus isolation and a seroconversion of anti-DENV IgM in paired serum samples. Serum samples with optical density (OD) absorbance value ≥2.0 in the in-house IgM capture enzyme-linked immunosorbent assay (ELISA) were detected as IgM-positive sera [62]. Serum samples were classified as either primary or secondary infection sera using the hemagglutination inhibition (HI) test [63]. Convalescent serum samples with an HI titer <1:1280 were classified as primary infection sera, whilst those with an HI titer ≥1:2560 were classified as secondary infection sera [64].

### Foci reduction neutralization test (FRNT)

Serum of convalescing dengue patient with primary infection with the respective DENV-1 genotype I and genotype II were used to determine their cross-neutralizing capacities against viruses from other subgroups. Due to the very limited amount of serum, serum samples (2–3 serum samples) from each of the different genotype group were pooled. The pooled serum was heat-inactivated at 56°C for 30 min and diluted four-fold (1:10 to 1:10240) in FBS-free EMEM medium. Serum samples (100 μl) were incubated with 100 FFU of virus (100 μl) at 37°C for 1 h. Subsequently, the mixtures (200 μl) were transferred to the C6/36 cell monolayer in 24-well plate and incubated at room temperature for 1 h. The mixtures were replaced with overlay EMEM medium [supplemented with 2% FBS and 1.5% carboxymethylcellulose (CMC)] and the plate was incubated at 28°C for 4 days. Foci of infected cells were visualized by using peroxidase-based foci staining assay as described previously [62,65]. The neutralizing antibody titer was expressed as the maximum serum dilution yielding 80% reduction in foci formed (FRNT_80_).

## Competing interests

The authors declare that they have no competing interests.

## Authors’ contributions

BTT and SSS analyzed and interpreted the data, performed the experiments, and wrote the manuscript. KKT, JJ, MHS, MBD, JAJ, and NAMR performed the virus propagation, amplification and sequencing of the viral envelop gene. NMM helped to analyze and interpret the data. SAB conceived and designed the study, coordinated the experiments, analyzed and interpreted the data, and wrote the manuscript. All authors have read and approved the final manuscript.
